# Artificial intelligence-enabled detection and assessment of Parkinson’s disease using nocturnal breathing signals

**DOI:** 10.1038/s41591-022-01932-x

**Published:** 2022-08-22

**Authors:** Yuzhe Yang, Yuan Yuan, Guo Zhang, Hao Wang, Ying-Cong Chen, Yingcheng Liu, Christopher G. Tarolli, Daniel Crepeau, Jan Bukartyk, Mithri R. Junna, Aleksandar Videnovic, Terry D. Ellis, Melissa C. Lipford, Ray Dorsey, Dina Katabi

**Affiliations:** 1 Department of Electrical Engineering and Computer Science, Massachusetts Institute of Technology, Cambridge, MA USA; 2 Department of Computer Science, Rutgers University, Piscataway, NJ USA; 3 Department of Neurology, University of Rochester Medical Center, Rochester, NY USA; 4 Center for Health and Technology, University of Rochester Medical Center, Rochester, NY USA; 5 Department of Neurology, Mayo Clinic, Rochester, MN USA; 6 Division of Cardiovascular Diseases, Mayo Clinic, Rochester, MN USA; 7 Department of Neurology and Center for Sleep Medicine, Division of Pulmonary and Critical Care Medicine, Mayo Clinic, Rochester, MN USA; 8 Divisions of Sleep Medicine and Movement Disorders, Massachusetts General Hospital, Boston, MA USA; 9 Department of Physical Therapy and Athletic Training, Center for Neurorehabilitation, Boston University College of Health and Rehabilitation, Sargent College, Boston, MA USA; 10 Emerald Innovations, Inc., Cambridge, MA USA

**Keywords:** Parkinson's disease, Parkinson's disease, Parkinson's disease, Parkinson's disease, Biomarkers

## Abstract

There are currently no effective biomarkers for diagnosing Parkinson’s disease (PD) or tracking its progression. Here, we developed an artificial intelligence (AI) model to detect PD and track its progression from nocturnal breathing signals. The model was evaluated on a large dataset comprising 7,671 individuals, using data from several hospitals in the United States, as well as multiple public datasets. The AI model can detect PD with an area-under-the-curve of 0.90 and 0.85 on held-out and external test sets, respectively. The AI model can also estimate PD severity and progression in accordance with the Movement Disorder Society Unified Parkinson’s Disease Rating Scale (*R* = 0.94, *P* = 3.6 × 10^–25^). The AI model uses an attention layer that allows for interpreting its predictions with respect to sleep and electroencephalogram. Moreover, the model can assess PD in the home setting in a touchless manner, by extracting breathing from radio waves that bounce off a person’s body during sleep. Our study demonstrates the feasibility of objective, noninvasive, at-home assessment of PD, and also provides initial evidence that this AI model may be useful for risk assessment before clinical diagnosis.

## Abstract (web summary)

Using a neural network-based model, Parkinson’s disease can be diagnosed and its severity monitored based on breathing patterns while someone is asleep, with the potential for at-home touchless monitoring.

## Main

PD is the fastest-growing neurological disease in the world^1^. Over 1 million people in the United States are living with PD as of 2020 (ref. ^2^), resulting in an economic burden of $52 billion per year^3^. Thus far, no drugs can reverse or stop the progression caused by the disease^4^. A key difficulty in PD drug development and disease management is the lack of effective diagnostic biomarkers^5^. The disease is typically diagnosed based on clinical symptoms, related mainly to motor functions such as tremor and rigidity^6^. However, motor symptoms tend to appear several years after the onset of the disease, leading to late diagnosis^4^. Thus, there is a strong need for new diagnostic biomarkers, particularly ones that can detect the disease at an early stage.

There are also no effective progression biomarkers for tracking the severity of the disease over time^5^. Today, assessment of PD progression relies on patient self-reporting or qualitative rating by a clinician^7^. Typically, clinicians use a questionnaire called the Movement Disorder Society Unified Parkinson’s Disease Rating Scale (MDS-UPDRS)^8^. The MDS-UPDRS is semisubjective and does not have enough sensitivity to capture small changes in patient status^9–11^. As a result, PD clinical trials need to last several years before changes in MDS-UPDRS can be reported with sufficient statistical confidence^9,12^, which increases cost and delays progress^13^.

The literature has investigated a few potential PD biomarkers, among which cerebrospinal fluid^14,15^, blood biochemical^16^ and neuroimaging^17^ have good accuracy. However, these biomarkers are costly, invasive and require access to specialized medical centers and, as a result, are not suitable for frequent testing to provide early diagnosis or continuous tracking of disease progression.

A relationship between PD and breathing was noted as early as 1817, in the work of James Parkinson^18^. This link was further strengthened in later work which reported degeneration in areas in the brainstem that control breathing^19^, weakness of respiratory muscle function^20^ and sleep breathing disorders^21–24^. Further, these respiratory symptoms often manifest years before clinical motor symptoms^20,23,25^, which indicates that the breathing attributes could be promising for risk assessment before clinical diagnosis.

**Fig. 1:**
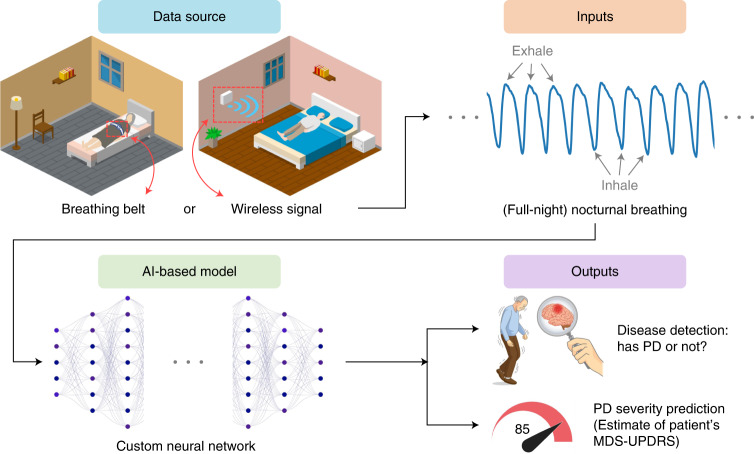
Overview of the AI model for PD diagnosis and disease severity prediction from nocturnal breathing signals. The system extracts nocturnal breathing signals either from a breathing belt worn by the subject, or from radio signals that bounce off their body while asleep. It processes the breathing signals using a neural network to infer whether the person has PD, and if they do, assesses the severity of their PD in accordance with the MDS-UPDRS.

**Extended Data Fig. 1:**
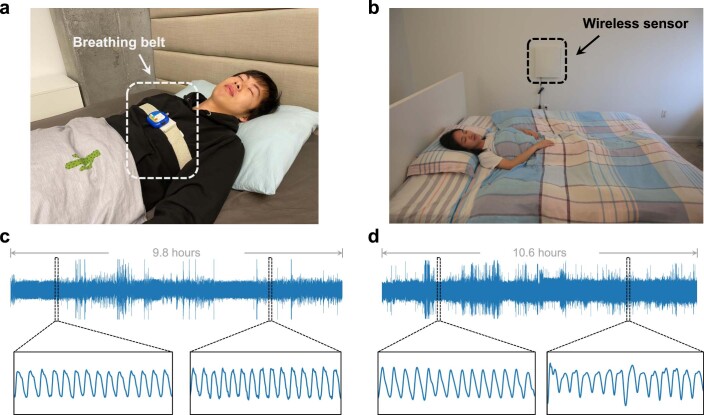
**Nocturnal breathing data collection setup.** **a**, Data from the breathing belt is collected by wearing an on-body breathing belt during sleep. **b**, Data from wireless signals is collected by installing a low-power wireless sensor in the subject’s bedroom, and extracting the subject’s breathing signals from the radio signals reflected off their body. **c, d**, Two samples of full-night nocturnal breathing from breathing belt and wireless signal and their zoomed-in versions.

In this article, we present a new AI-based system (Fig. 1 and Extended Data Fig. 1) for detecting PD, predicting disease severity and tracking disease progression over time using nocturnal breathing. The system takes as input one night of breathing signals, which can be collected using a breathing belt worn on the person’s chest or abdomen^26^. Alternatively, the breathing signals can be collected without wearable devices by transmitting a low power radio signal and analyzing its reflections off the person’s body^27–29^. An important component of the design of this model is that it learns the auxiliary task of predicting the person’s quantitative electroencephalogram (qEEG) from nocturnal breathing, which prevents the model from overfitting and helps in interpreting the output of the model. Our system aims to deliver a diagnostic and progression digital biomarker that is objective, nonobtrusive, low-cost and can be measured repeatedly in the patient’s home.

## Results

### Datasets and model training

We use a large and diverse dataset created by pulling several datasets from several sources, including the Mayo Clinic, Massachusetts General Hospital (MGH) sleep lab, observational PD clinical trials sponsored by the Michael J. Fox Foundation (MJFF) and the National Institutes of Health (NIH) Udall Center, an observational study conducted by the Massachusetts Institute of Technology (MIT) and public sleep datasets from the National Sleep Research Resource such as the Sleep Heart Health Study (SHHS)^26^ and the MrOS Sleep Study (MrOS)^30^. The combined dataset contains 11,964 nights with over 120,000 h of nocturnal breathing signals from 757 PD subjects (mean (s.d.) age 69.1 (10.4), 27% women) and 6,914 control subjects (mean (s.d.) age 66.2 (18.3), 30% women). Table 1 summarizes the datasets and Extended Data Table 1 describes their demographics.

**Table 1:** Characteristics of the datasets used in this study

Data source	Data type	Source of breathing signals	No. of PD patients	No. of controls	MDS-UPDRS	H&Y stage	No. of nights per subject
Mayo Clinic (External test cohort)	PSG sleep study (sampled from the population visiting the Mayo Clinic sleep lab)	Breathing belt	644	1,276	–	PD: 2.2 (1.0) Control: N/A	1 (0)
SHHS Visit 2	PSG sleep study (heart disease, sleep disorders)	Breathing belt	13	2,617	–	–	1 (0)
MrOS Sleep Study	PSG sleep study (sleep disorders, vascular disease)	Breathing belt	48	2,827	–	–	1.4 (0.5)
MGH study	PSG sleep study (sampled from the population visiting the MGH sleep lab)	Breathing belt	27	120	PD: 39.8 (17.4) Control: N/A	PD: 2.2 (0.4) Control: N/A	1 (0)
MGH study	Sleep study (sampled from the population visiting the MGH sleep lab)	Wireless	0	8	N/A	N/A	9.5 (4.0)
Udall study	Observational clinical study in PD	Wireless	14	6	PD: 61.1 (20.1) Control: 1.8 (2.0)	PD: 2.3 (0.6) Control: 0.2 (0.4)	86.7 (67.2)
MJFF Parkinson’s study	Observational clinical study in PD	Wireless	11	4	PD: 58.3 (19.3) Control: 7.0 (1.9)	PD: 2.2 (0.7) Control: 0 (0)	35.1 (19.1)
MIT study	Sleep study (healthy volunteers)	Wireless	0	56	N/A	N/A	18.7 (24.4)

Dashes, unavailable data; N/A, inapplicable data.

The data were divided into two groups: the breathing belt datasets and the wireless datasets. The first group comes from polysomnography (PSG) sleep studies and uses a breathing belt to record the person’s breathing throughout the night. The second group collects nocturnal breathing in a contactless manner using a radio device^27^. The radio sensor is deployed in the person’s bedroom, and analyzes the radio reflections from the environment to extract the person’s breathing signal^28,29^.

The breathing belt datasets have only one or two nights per person and lack MDS-UPDRS and Hoehn and Yahr (H&Y) scores^32^. In contrast, the wireless datasets include longitudinal data for up to 1 year and MDS-UPDRS and H&Y scores, allowing us to validate the model’s predictions of PD severity and its progression. Since some individuals in the wireless datasets are fairly young (for example, in their 20s or 30s), when testing on the wireless data, we limit ourselves to the PD patients and their age-matched control subjects (that is, 10 control subjects from the Udall and MJFF studies and 18 age and gender-matched subjects from the MIT and MGH studies for a total of 28 control individuals). Control subjects missing MDS-UPDRS or H&Y scores receive the mean value for the control group.

Subjects used in training the neural network were not used for testing. We performed *k*-fold cross-validation (*k* = 4) for PD detection, and leave-one-out validation for severity prediction. We also assessed cross-institution prediction by training and testing the model on data from different medical centers. Furthermore, data from the Mayo Clinic was kept as external data, never seen during development or validation, and used only for a final test.

### Evaluation of PD diagnosis

**Fig. 2:**
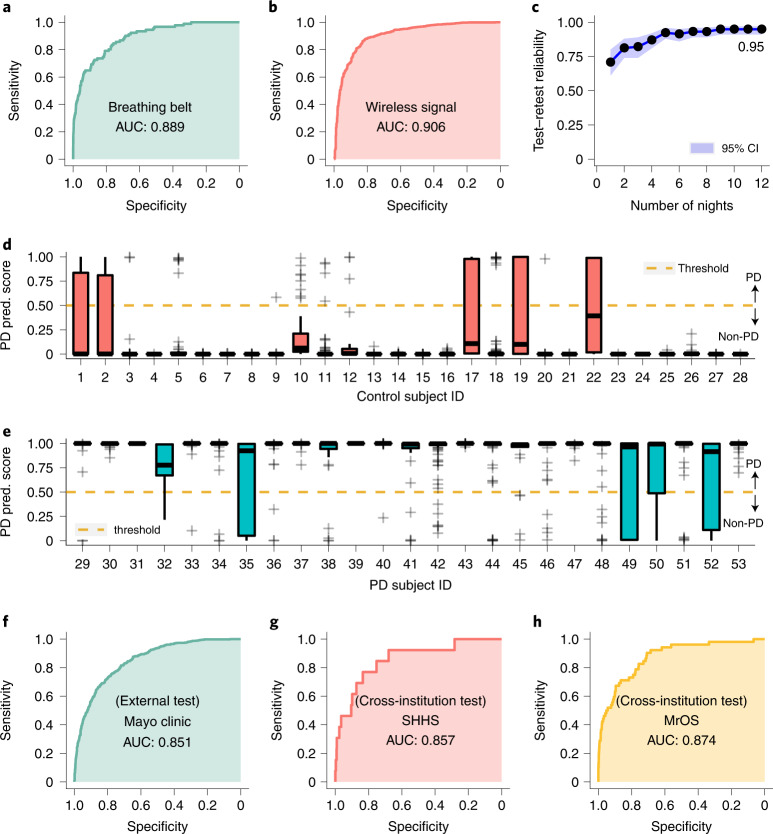
PD diagnosis from nocturnal breathing signals. **a**, ROC curves for detecting PD from breathing belt (*n* = 6,660 nights from 5,652 subjects). **b**, ROC curves for detecting PD from wireless data (*n* = 2,601 nights from 53 subjects). **c**, Test–retest reliability of PD diagnosis as a function of the number of nights used by the AI model. The test was performed on 1 month of data from each subject in the wireless dataset (*n* = 53 subjects). The dots and the shadow denote the mean and 95% CI, respectively. The model achieved a reliability of 0.95 (95% CI (0.92, 0.97)) with 12 nights of data. **d**,**e**, Distribution of PD prediction (pred.) scores for subjects with several nights (*n*_1_ = 1,263 nights from 25 PD subjects and *n*_2_ = 1,338 nights from 28 age- and gender-matched controls). The graphs show a boxplot of the prediction scores as a function of the subject ids. On each box, the central line indicates the median, and the bottom and top edges of the box indicate the 25th and 75th percentiles, respectively. The whiskers extend to 1.5 times the interquartile range. Points beyond the whiskers are plotted individually using the + symbol. **f**, ROC curves for detecting PD on an external test set from Mayo Clinic (*n* = 1,920 nights from 1,920 subjects). The model has an AUC of 0.851 with a sensitivity of 80.12% and specificity of 72.65%. **g**, Cross-institution PD prediction performance on SHHS (*n* = 2,630 nights from 2,630 subjects). **h**, Cross-institution PD prediction performance on MrOS (*n* = 3,883 nights from 2,875 subjects). In this analysis, all data from one institution was held back as test data, and the AI model was retrained excluding all data from that institution. Cross-institution prediction achieved an AUC of 0.857 with a sensitivity of 76.92% and specificity of 83.45% on SHHS, and an AUC of 0.874 with a sensitivity of 82.69% and specificity of 75.72% on MrOS.

**Extended Data Fig. 2:**
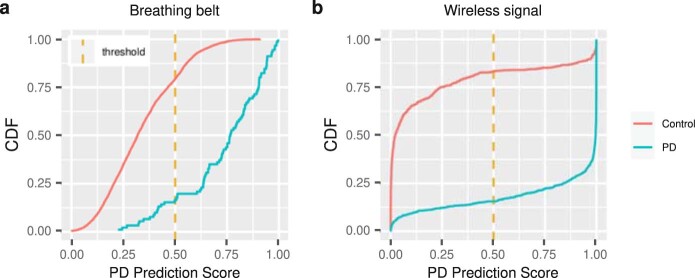
Cumulative distributions of the prediction score for PD diagnosis. **a**, Results for breathing belt data (n = 6,660 nights from 5,652 subjects). **b**, Results for wireless data (n = 2,601 nights from 53 subjects). For both data types, fixing a threshold of 0.5 leads to good performance (that is, sensitivity 80.22% and specificity 78.62% for breathing belt, and sensitivity 86.23% and specificity 82.83% for wireless data).

We evaluated the accuracy of diagnosing PD from one night of nocturnal breathing. Figure 2a,b show the receiver operating characteristic (ROC) curves for data from breathing belt and data from wireless signals, respectively. The AI model detects PD with high accuracy. For nights measured using a breathing belt, the model achieves an area under the ROC curve (AUC) of 0.889 with a sensitivity of 80.22% (95% confidence interval (CI) (70.28%, 87.55%)) and specificity of 78.62% (95% CI (77.59%, 79.61%)). For nights measured using wireless signals, the model achieves an AUC of 0.906 with a sensitivity of 86.23% (95% CI (84.08%, 88.13%)) and specificity of 82.83% (95% CI (79.94%, 85.40%)). Extended Data Fig. 2 further shows the cumulative distributions of the prediction score for PD diagnosis.

We further investigated whether the accuracy improves by combining several nights from the same individual. We use the wireless datasets since they have several nights per subject (mean (SD) 61.3 (42.5)), and compute the model prediction score for all nights. The PD prediction score is a continuous number between 0 and 1, where the subject is considered to have PD if the score exceeds 0.5. We use the median PD score for each subject as the final diagnosis result. As shown in Fig. 2d,e, with several nights considered for each subject, both sensitivity and specificity of PD diagnosis further increase to 100% for the PD and control subjects in this cohort.

Next, we compute the number of nights needed to achieve a high test–retest reliability^31^. We use the wireless datasets, and compute the test–retest reliability by averaging the prediction across consecutive nights within a time window. The results show that the reliability improves when we use several nights from the same subject, and reaches 0.95 (95% CI (0.92, 0.97)) with only 12 nights (Fig. 2c).

### Generalization to external test cohort

To assess the generalizability of our model across different institutions with different data collection protocols and patient populations, we validated our AI model on an external test dataset (*n* = 1,920 nights from 1,920 subjects out of which 644 have PD) from an independent hospital not involved during model development (Mayo Clinic). Our model achieved an AUC of 0.851 (Fig. 2f). The performance indicates that our model can generalize to diverse data sources from institutions not encountered during training.

We also examined the cross-institution prediction performance by testing the model on data from one institution, but training it on data from the other institutions excluding the test institution. For breathing belt data, and as highlighted in Fig. 2g,h, the model achieved a cross-institution AUC of 0.857 on SHHS and 0.874 on MrOS. For wireless data, the cross-institution performance was 0.892 on MJFF, 0.884 on Udall, 0.974 on MGH and 0.916 on MIT. These results show that the model is highly accurate on data from institutions it never saw during training. Hence, the accuracy is not due to leveraging institution-related information, or misattribution of institution-related information to the disease.

### Evaluation of PD severity prediction

Today the MDS-UPDRS is the most common method for evaluating PD severity, with higher scores indicating more severe impairment. Evaluating MDS-UPDRS requires effort from both patients and clinicians: patients are asked to visit the clinic in person and evaluations are performed by trained clinicians who categorize symptoms based on quasi-subjective criteria^9^.

**Fig. 3:**
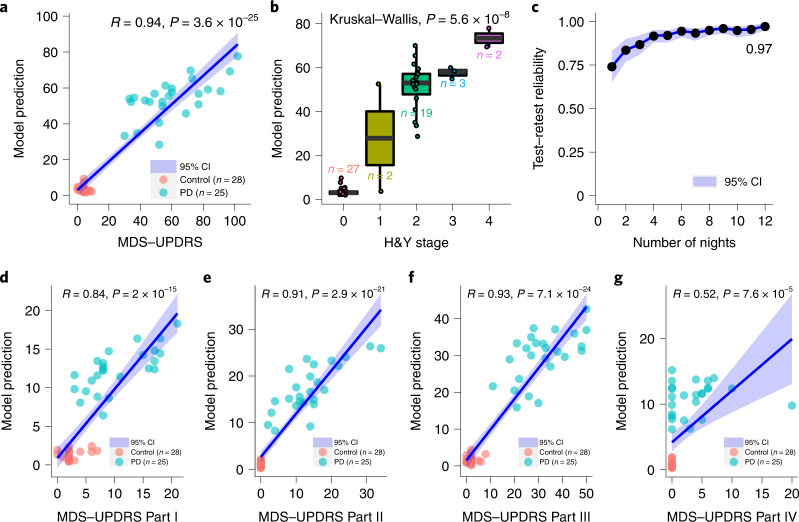
PD severity prediction from nocturnal breathing signals. **a**, Severity prediction of the model with respect to MDS-UPDRS (two-sided *t*-test). The center line and the shadow denote the mean and 95% CI, respectively. **b**, Severity prediction distribution of the model with respect to the H&Y stage; a higher H&Y stage indicates increased PD severity (Kruskal–Wallis test). On each box, the central line indicates the median, and the bottom and top edges of the box indicate the 25th and 75th percentiles, respectively. The whiskers extend to 1.5 times the interquartile range. **c**, Test–retest reliability of PD severity prediction as a function of the number of nights per subject. The dots and the shadow denote the mean and 95% CI, respectively. The model achieved a reliability of 0.97 (95% CI (0.95, 0.98)) with 12 nights of data. **d**, Correlation of the AI model predictions with MDS-UPDRS subpart I (two-sided *t*-test). **e,** Correlation of the AI model predictions with MDS-UPDRS subpart II (two-sided *t*-test). **f,** Correlation of the AI model predictions with MDS-UPDRS subpart III (two-sided *t*-test). **g,** Correlation of the AI model predictions MDS-UPDRS subpart IV (two-sided *t*-test). The center line and the shadow denote the mean and 95% CI, respectively. Data in all panels are from the wireless dataset (*n* = 53 subjects).

We evaluate the ability of our model to produce a PD severity score that correlates well with the MDS-UPDRS simply by analyzing the patients’ nocturnal breathing at home. We use the wireless dataset where MDS-UPDRS assessment is available, and each subject has several nights of measurements (*n* = 53 subjects, 25 PD subjects with a total of 1,263 nights and 28 controls with a total of 1,338 nights). We compare the MDS-UPDRS at baseline with the model’s median prediction computed over the nights from the 1-month period following the subject’s baseline visit. Figure 3a shows strong correlation between the model’s severity prediction and the MDS-UPDRS (*R* = 0.94, *P* = 3.6 × 10^–25^), providing evidence that the AI model can capture PD disease severity.

We also studied the feasibility of predicting each of the four subparts of MDS-UPDRS (that is, predicting subparts I, II, III and IV). This was done by replacing the module for predicting the total MDS-UPDRS by a module that focuses on the subpart of interest, while keeping all the other components of the neural network unmodified. Figure 3d–g show the correlation between the model prediction and the different subparts of MDS-UPDRS. We observe a strong correlation between model prediction and Part I (*R* = 0.84, *P* = 2 × 10^–15^), Part II (*R* = 0.91, *P* = 2.9 × 10^–21^) and Part III (*R* = 0.93, *P* = 7.1 × 10^–24^) scores. This indicates that the model captures both nonmotor (for example, Part I), and motor symptoms (for example, Part II and III) of PD. The model’s prediction has mild correlation with Part IV (*R* = 0.52, *P* = 7.6 × 10^–5^). This may be caused by the large overlap between PD and control subjects in Part IV scores (that is, most of the PD patients and control subjects in the studied population have a score of 0 for Part IV).

We also compared the severity prediction of our model with H&Y stage^32^—another standard for PD severity estimation. The H&Y stage uses a categorical scale, where a higher stage indicates worse severity. Again, we used the Udall and the MJFF datasets since they report the H&Y scores and have several nights per subject. Figure 3b shows that, even though it is not trained using H&Y, the model can differentiate patients reliably in terms of their H&Y stages (*P* = 5.6 × 10^–8^, Kruskal–Wallis test).

Finally, we computed the test–retest reliability of PD severity prediction on the same datasets in Fig. 3c. Our model provides consistent and reliable predictions for assessing PD severity with its reliability reaching 0.97 (95% CI (0.95, 0.98)) with 12 nights per subject.

### PD risk assessment

**Fig. 4:**
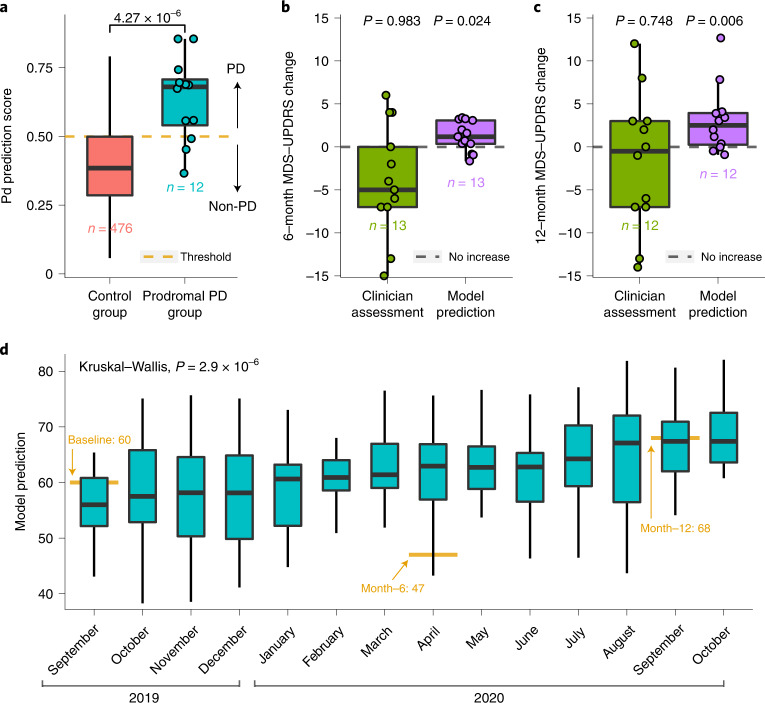
Model evaluation for PD risk assessment before actual diagnosis, and disease progression tracking using longitudinal data. **a**, Model prediction scores for the prodromal PD group (that is, undiagnosed individuals who were eventually diagnosed with PD) and the age- and gender-matched control group (one-tailed Wilcoxon rank-sum test). **b**, The AI model assessment of the change in MDS-UPDRS over 6 months (one-tailed one-sample Wilcoxon signed-rank test) and the clinician assessment of the change in MDS-UPDRS over the same period (one-tailed one-sample Wilcoxon signed-rank test). **c**, The AI model assessment of the change in MDS-UPDRS over 12 months (one-tailed one-sample Wilcoxon signed-rank test) and the clinician assessment of the change in MDS-UPDRS over the same period (one-tailed one-sample Wilcoxon signed-rank test). **d**, Continuous severity prediction across 1 year for the patient with maximum MDS-UPDRS increase (Kruskal–Wallis test; *n* = 365 nights from 1 September 2019 to 31 October 2020). For each box in **a**–**d**, the central line indicates the median, and the bottom and top edges of the box indicate the 25th and 75th percentiles, respectively. The whiskers extend to 1.5 times the interquartile range.

Since breathing and sleep are impacted early in the development of PD^4,23,25^, we anticipate that our AI model can potentially recognize individuals with PD before their actual diagnosis. To evaluate this capability, we leveraged the MrOS dataset^30^, which includes breathing and PD diagnosis from two different visits, separated by approximately 6 years. We considered subjects who were diagnosed with PD by their second visit, but had no such diagnosis by their first visit, and refer to them as the ‘prodromal PD group’ (*n* = 12). To select the ‘control group’, we sample subjects from the MrOS dataset who did not have a PD diagnosis in the first visit or in the second visit, occurring 6 years later. For each of the subjects in the prodromal group, we sample up to 40 control subjects that are age- and gender-matched, resulting in 476 qualified control subjects. We evaluated our model on breathing data from the first visit, when neither the prodromal group nor the control group had a PD diagnosis. Figure 4a shows that the model gives the prodromal group (that is, subjects eventually diagnosed with PD) much higher PD scores than the control group (*P* = 4.27 × 10^–6^, one-tailed Wilcoxon rank-sum test). Indeed, the model predicts 75% of them as individuals with PD before their reported PD diagnosis.

### PD disease progression

Today, assessment of PD progression relies on MDS-UPDRS, which is semisubjective and does not have enough sensitivity to capture small, progressive changes in patient status^9,10^. As a result, PD clinical trials need to last for several years before changes in MDS-UPDRS can be reported with sufficient statistical confidence^9,12^, which creates a great challenge for drug development. A progression marker that captures statistically significant changes in disease status over short intervals could shorten PD clinical trials.

We evaluated disease progression tracking on data from the Udall study, which includes longitudinal data from participants with PD 6 months (*n* = 13) and 12 months (*n* = 12) into the study. For those individuals, we assess their disease progression using two methods. In the first method, we use the difference in the clinician-scored MDS-UPDRS at baseline and at month 6, or month 12. In the second method, we use the change in their predicted MDS-UPDRS over 6 months or 12 months. To compute the change in the predicted MDS-UPDRS, we took the data from the 1 month following baseline and computed its median MDS-UPDRS prediction, and the month following the month-6 visit and computed its median MDS-UPDRS prediction. We then subtracted the median at month 6 from the median at baseline. We repeated the same procedure for computing the prediction difference between month 12 and baseline. We plotted the results in Fig. 4b,c. The results show both the 6-month and 1-year changes in MDS-UPDRS as scored by a clinician are not statistically significant (6-month *P* = 0.983, 12-month *P* = 0.748, one-tailed one-sample Wilcoxon signed-rank test), which is consistent with previous observations^9,10,12^. In contrast, the model’s estimates of changes in MDS-UPDRS over the same periods are statistically significant (6-month *P* = 0.024, 12-month *P* = 0.006, one-tailed one-sample Wilcoxon signed-rank test).

**Extended Data Fig. 3:**
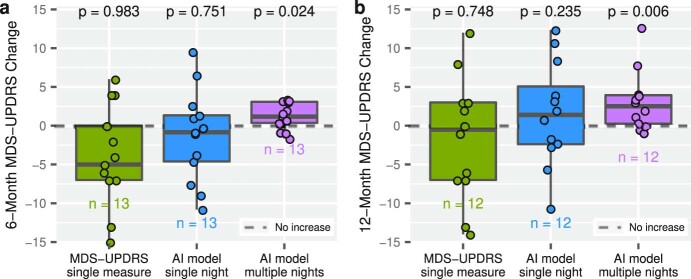
Disease progression tracking using a different number of nights. **a, b**, 6-month and 12-month change in MDS-UPDRS as assessed by a clinician and predicted by the AI model, both using a single night and multiple nights of data. On each box, the central line indicates the median, and the bottom and top edges of the box indicate the 25th and 75th percentiles, respectively. The whiskers extend to 1.5 times the interquartile range. Similar to the clinician assessment, when using only a single night of data, the AI model cannot detect statistically significant changes over a year (p = 0.751 for 6 months, p = 0.235 for 12 months, one-tailed one-sample Wilcoxon signed-rank test). This indicates that the reason why the AI model can achieve statistical significance for progression analysis while MDS-UPDRS cannot stems from being able to combine measurements from multiple nights, which substantially reduces measurement noise and increases sensitivity.

The key reason why our model can achieve statistical significance for progression analysis while the clinician-scored MDS-UPDRS cannot stems from being able to aggregate measurements from several nights. Any measurement, whether the clinician-scored MDS-UPDRS or the model-predicted MDS-UPDRS, has some noise. By aggregating a large number of measurements, one can reduce the noise and improve sensitivity to disease progression over a short period. This is feasible for the model-predicted MDS-UPDRS because the measurements can be repeated every night with no overhead to patients. In contrast, one cannot do the same for the clinician-scored MDS-UPDRS as it is infeasible to ask the patient to come to the clinic every day to repeat the MDS-UPDRS test. This point is illustrated in Extended Data Fig. 3, which shows that, if the model-predicted MDS-UPDRS used a single night for tracking progression, then, similarly to clinician-scored MDS-UPDRS, it would be unable to achieve statistical significance.

To provide more insight, we examined continuous severity tracking over 1 year for the patient in our cohort who exhibited the maximum increase in MDS-UPDRS over this period (Fig. 4d). The results show that the AI model can achieve statistical significance in tracking disease progression in this patient from one month to the next (*P* = 2.9 × 10^–6^, Kruskal–Wallis test). The figure also shows that the clinician-scored MDS-UPDRS is noisy; the MDS-UPDRS at month 6 is lower than at baseline, although PD is a progressive disease and the severity should be increasing monotonically.

Finally, we note that the above results persist if one controls for changes in symptomatic therapy. Specifically, we repeated the above analysis, limiting it to patients who had no change in symptomatic therapy. The changes in the model-predicted MDS-UPDRS are statistically significant (6-month *P* = 0.049, 12-month *P* = 0.032, one-tailed one-sample Wilcoxon signed-rank test), whereas the changes in the clinician-scored MDS-UPDRS are statistically insignificant (6-month *P* = 0.894, 12-month *P* = 0.819, one-tailed one-sample Wilcoxon signed-rank test).

### Distinguishing PD from Alzheimer’s disease

**Extended Data Fig. 4:**
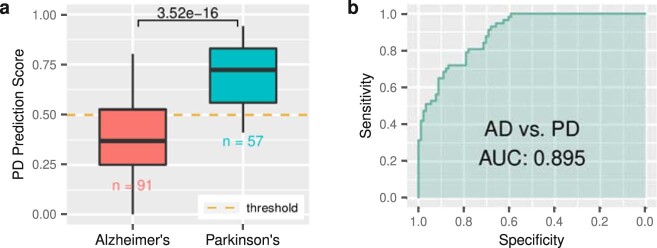
Performance of the AI model on differentiating subjects with Parkinson’s disease (PD) from subjects with Alzheimer’s disease (AD). **a**, The model’s output scores differentiate PD subjects from AD subjects (p = 3.52e-16, one-tailed Wilcoxon rank-sum test). **b**, Receiver operating characteristic (ROC) curves for detecting PD subjects against AD subjects (n = 148). The model achieves high AUC for differentiating PD from AD (AUC = 0.895).

We additionally tested the ability of the model to distinguish between PD and Alzheimer’s disease (AD)—the two most common neurodegenerative diseases. To evaluate this capability, we leveraged the SHHS^26^ and MrOS^30^ datasets, which contain subjects identified with AD (Methods). In total, 99 subjects were identified with AD, with 9 of these also reported to have PD. We excluded subjects with both AD and PD, and evaluate the ability of our model to distinguish the PD group (*n* = 57) from the AD group (*n* = 91). Extended Data Fig. 4 shows that the model achieves an AUC of 0.895 with a sensitivity of 80.70% and specificity of 78.02% in differentiating PD from AD, and reliably distinguished PD from AD subjects (*P* = 3.52 × 10^–16^, one-tailed Wilcoxon rank-sum test).

### Model interpretability

Our AI model employs a self-attention module^33^, which scores each interval of data according to its contribution to making a PD or non-PD prediction (model details in Methods). Since the SHHS and MrOS datasets include EEG signals and sleep stages throughout the night, we can analyze the breathing periods with high attention scores, and the corresponding sleep stages and EEG bands. Such analysis allows for interpreting and explaining the results of the model.

**Fig. 5:**
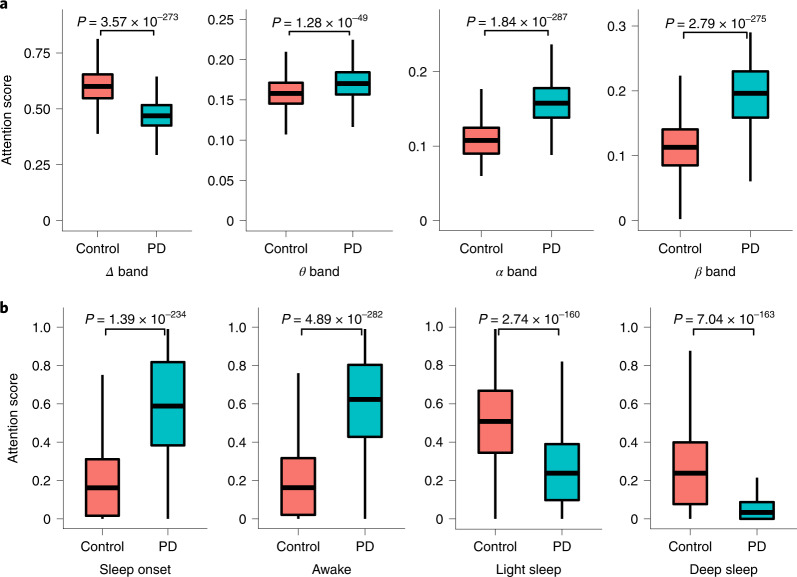
Interpretation of the output of the AI model with respect to EEG and sleep status. **a**,**b**, Attention scores were aggregated according to sleep status and EEG bands for PD patients (*n* = 736 nights from 732 subjects) and controls (*n* = 7,844 nights from 6,840 subjects). Attention scores were normalized across EEG bands or sleep status. Attention scores for different EEG bands between PD patients and control individuals (one-tailed Wilcoxon rank-sum test) (**a**). Attention scores for different sleep status between PD patients and control individuals (one-tailed Wilcoxon rank-sum test) (**b**). On each box in **a** and **b**, the central line indicates the median, and the bottom and top edges of the box indicate the 25th and 75th percentiles, respectively. The whiskers extend to 1.5 times the interquartile range.

**Extended Data Fig. 5:**
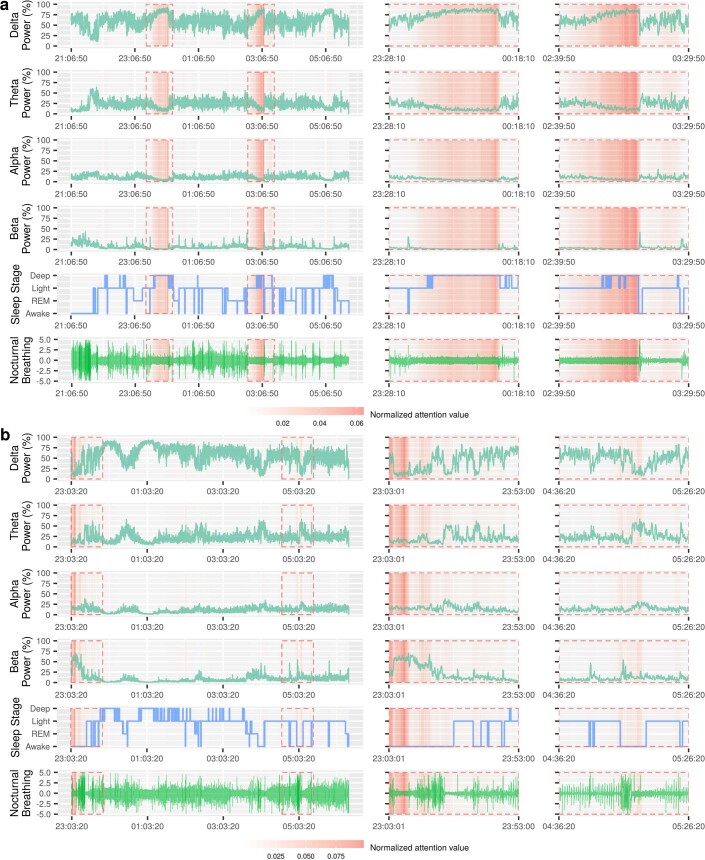
Visualization examples of the attention of the AI model. **a, b**, Full-night attention distribution (left) and its zoomed-in version (right) overlayed on the corresponding qEEG bands, sleep stages, and breathing. Graphs show a control subject in (a) and a PD patient in (b). For the control subject, the model’s attention focuses on periods with high Delta waves, which correspond to deep sleep. In contrast, for the PD subject, the model attends to periods with relatively high Beta or Alpha waves, and awakenings around sleep onset and in the middle of sleep.

The analysis shows that the attention of the model focuses on periods with relatively high qEEG Delta activity for control individuals, while focusing on periods with high activities in *β* and other bands for PD patients (Fig. 5a). Interestingly, these differences are aligned with previous work that observed that PD patients have reduced power in Delta band and increased power in *β* and other EEG bands during non-REM (rapid eye movement) sleep^34,35^. Further, comparing the model’s attention to the person’s sleep stages shows that the model recognizes control subjects by focusing on their light/deep sleep periods, while attending more to sleep onset and awakenings in PD patients (Fig. 5b). This is consistent with the medical literature, which reports that PD patients have substantially less light and deep sleep, and more interruptions and wakeups during sleep^34,36^, and the EEG in PD patients during sleep onset and awake periods show abnormalities in comparison with non-PD individuals^37–39^. (For more insight, Extended Data Fig. 5 shows a visualization of the attention score for one night, and the corresponding sleep stages and qEEG for a PD and a control individual.)

### Subanalyses and ablation studies

#### Performance dependence on model subcomponents

**Extended Data Fig. 6:**
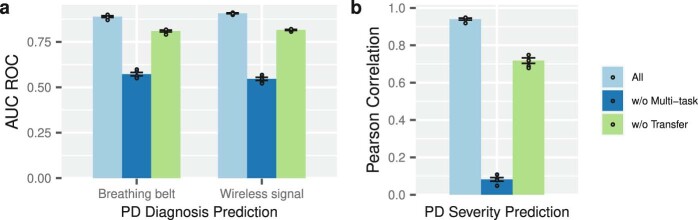
Ablation studies for assessing the benefit of multi-task learning and transfer learning. **a**, PD diagnosis performance on breathing belt data (n = 6,660 nights from 5,652 subjects) and wireless data (n = 2,601 nights from 53 subjects), for the model with all of its components and the model without the qEEG auxiliary task (that is, without multi-task learning) and without transfer learning. Each bar and its error bar indicate the mean and standard deviation across 5 independent runs. The graphs indicate that the qEEG auxiliary task is essential for good performance and eliminating it reduces the AUC by almost 40%. Transfer learning also boosts performance for both breathing belt data (7.8% improvements) and wireless data (8.3% improvements), yet is not as essential as multi-task learning. **b**, Pearson correlation of the PD severity prediction and MDS-UPDRS. The correlation is computed for subjects in the wireless datasets (n = 53 subjects) since their MDS-UPDRS scores are available. Each bar and its error bar indicate the mean and standard deviation across 5 independent runs. The results indicate that transfer learning is useful, but multi-task learning (that is, the qEEG auxiliary task) is essential for good performance.

Our AI model employs multitask learning (that is, uses an auxiliary task of predicting qEEG) and transfer learning (Methods). We conducted ablation experiments to assess the benefits of (1) the qEEG auxiliary task, and (2) the use of transfer learning. To do so, we assessed the AUC of the model with and without each of those components. The results show that the qEEG auxiliary task is essential for good AUC, and transfer learning further improves the performance (Extended Data Fig. 6).

#### Comparison with machine learning baselines

**Extended Data Fig. 7:**
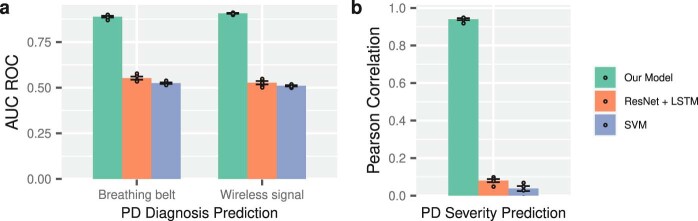
Performance comparison of the model with two machine learning baselines: Support Vector Machine (SVM) and a neural network based on ResNet and LSTM. **a**, PD diagnosis performance on breathing belt data (n = 6,660 nights from 5,652 subjects) and wireless data (n = 2,601 nights from 53 subjects). Each bar and its error bar indicate the mean and standard deviation across 5 independent runs. **b**, Pearson correlation of PD severity prediction and MDS-UPDRS. The correlation is computed for subjects in the wireless datasets (n = 53 subjects) since their MDS-UPDRS scores are available. Each bar and its error bar indicate the mean and standard deviation across 5 independent runs.

We compared the performance of our model with that of two machine learning models: a support vector machine (SVM)^40^ model, and a basic neural network that employs ResNet and LSTM but lacks our transfer learning module and the qEEG auxiliary task^41^. (More details about these baselines are provided in Methods.) The results show that both SVM and the ResNet+LSTM network substantially underperform our model (Extended Data Fig. 7).

#### Performance for different disease durations

We examined the accuracy of the model for different disease durations. We considered the Udall dataset, where disease duration was collected for each PD patient. We divided the patients into three groups based on their disease duration: less than 5 years (*n* = 6), 5 to 10 years (*n* = 4) and over 10 years (*n* = 4). The PD detection accuracy using one night per patient for these groups is: 86.5%, 89.4% and 93.9%, respectively. The PD detection accuracy increases to 100% for all three groups when taking the median prediction over 1 month. The errors in predicting the MDS-UPDRS for the three groups were 9.7%, 7.6% and 13.8%, respectively. These results show that the model has good performance across a wide range of disease durations.

### Evaluation of qEEG prediction from nocturnal breathing

**Extended Data Fig. 8:**
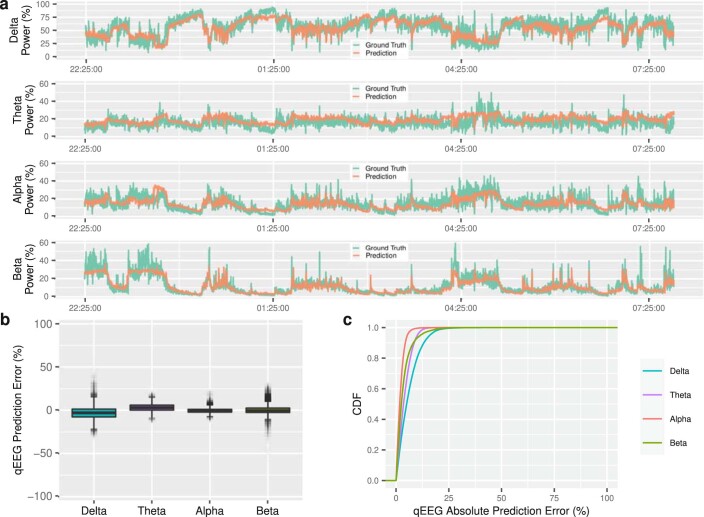
Evaluation results for predicting the full-night qEEG summary from nocturnal breathing signals. **a**, One prediction sample of a full-night qEEG. The time resolution of the predicted qEEG is 1 second. **b**, Distribution of the prediction errors across four EEG bands (n = 6,660 nights from 5,652 subjects). The AI model made an unbiased (that is, median-unbiased) estimation of EEG prediction for all bands. On each box, the central line indicates the median, and the bottom and top edges of the box indicate the 25th and 75th percentiles, respectively. The whiskers extend to 1.5 times the interquartile range. **c**, Cumulative distribution functions of the absolute prediction error across four EEG bands (n = 6,660 nights from 5,652 subjects).

Finally, since our model predicts qEEG from nocturnal breathing as an auxiliary task, we evaluate the accuracy of qEEG prediction. We use the SHHS, MrOS and MGH datasets, which include nocturnal EEG. The results show that our prediction can track the ground-truth power in different qEEG bands with good accuracy (Extended Data Fig. 8 and Supplementary Fig. 1).

## Discussion

This work provides evidence that AI can identify people who have PD from their nocturnal breathing and can accurately assess their disease severity and progression. Importantly, we were able to validate our findings in an independent external PD cohort. The results show the potential of a new digital biomarker for PD. This biomarker has several desirable properties. It operates both as a diagnostic (Figs. 2 and 3) and a progression (Fig. 4d) biomarker. It is objective and does not suffer from the subjectivity of either patient or clinician (Fig. 4b,c). It is noninvasive and easy to measure in the person’s own home. Further, by using wireless signals to monitor breathing, the measurements can be collected every night in a touchless manner.

Our results have several implications. First, our approach has the potential of reducing the cost and duration of PD clinical trials, and hence facilitating drug development. The average cost and time of PD drug development are approximately $1.3 billion and 13 years, respectively, which limits the interest of many pharmaceutical companies in pursuing new therapies for PD^13^. PD is a disease that progresses slowly and current methods for tracking disease progression are insensitive and cannot capture small changes^9,10,12,13^; hence, they require several years to detect progression^9,10,12,13^. In contrast, our AI-based biomarker has shown potential evidence of increased sensitivity to progressive changes in PD (Fig. 4d). This can help shorten clinical trials, reduce cost and speed up progress. Our approach can also improve patient recruitment and reduce churn because measurements can be collected at home with no overhead to patients.

Second, about 40% of individuals with PD currently do not receive care from a PD specialist^42^. This is because PD specialists are concentrated in medical centers in urban areas, while patients are spread geographically, and have problems traveling to such centers due to old age and limited mobility. By providing an easy and passive approach for assessing disease severity at home and tracking changes in patient status, our system can reduce the need for clinic visits and help extend care to patients in underserved communities.

Third, our system could also help in early detection of PD. Currently, diagnosis of PD is based on the presence of clinical motor symptoms^6^, which are estimated to develop after 50–80% of dopaminergic neurons have already degenerated^43^. Our system shows initial evidence that it could potentially provide risk assessment before clinical motor symptoms (Fig. 4a).

We envision that the system could eventually be deployed in the homes of PD patients and individuals at high risk for PD (for example, those with *LRRK2* gene mutation) to passively monitor their status and provide feedback to their provider. If the model detects severity escalation in PD patients, or conversion to PD in high-risk individuals, the clinician could follow up with the patient to confirm the results either via telehealth or a visit to the clinic. Future research is required to establish the feasibility of such use pattern, and the potential impact on clinical practice.

Our study also has some limitations. PD is a nonhomogeneous disease with many subtypes^44^. We did not explore subtypes of PD and whether our system works equally well with all subtypes. Another limitation of the paper is that both the progression analysis and preclinical diagnosis were validated in a small number of participants. Future studies with larger populations are required to further confirm those results. Also, while we have confirmed that our system could separate PD from AD, we did not investigate the ability of our model to separate PD from broader neurological diseases. Further, while we have tested the model across institutions and using independent datasets, further studies can expand the diversity of datasets and institutions. Additionally, our empirical results highlight a strong connection between PD and breathing and confirm past work on the topic; however, the mechanisms that lead to the development and progression of respiratory symptoms in PD are only partially understood and require further study.

Finally, our work shows that advances in AI can support medicine by addressing important unsolved challenges in neuroscience research and allowing for the development of new biomarkers. While the medical literature has reported several PD respiratory symptoms, such as weakness of respiratory muscles^20^, sleep breathing disorders^21–24^ and degeneration in the brain areas that control breathing^19^, without our AI-based model, no physician today can detect PD or assess its severity from breathing. This shows that AI can provide new clinical insights that otherwise may be inaccessible.

## Methods

### Dataset descriptions

Additional information about the datasets used in this study are summarized in Table 1 and their demographics are provided in Extended Data Table 1.

#### SHHS dataset

The SHHS^26^ dataset (visit 2) is a multicenter cohort PSG study of cardiovascular diseases and sleep-related breathing. Dataset description and ethical oversight are available at the National Sleep Research Resource (https://sleepdata.org/datasets/shhs).

#### MrOS dataset

The MrOS^30^ dataset is a multicenter cohort PSG study for understanding the relationship between sleep disorders and falls, fractures, mortality and vascular disease. The dataset description and ethical oversight are available at the National Sleep Research Resource (https://sleepdata.org/datasets/mros).

#### Udall dataset

The Udall dataset is comprised of PD and normal participants who underwent an in-home observational study between 1 June 2019 and 1 January 2021. Inclusion criteria require participants to be at least 30 years of age, able and willing to provide informed consent, have Wi-Fi in their residence, have English fluency and be resident in the United States. Exclusion criteria include nonambulatory status, pregnancy, having more than one ambulatory pet in the household, the inability to complete study activities as determined by the study team and any medical or psychiatric condition that, in the study team’s judgment, would preclude participation. Additionally, the PD participants were required to have a diagnosis of PD according to the UK PD Brain Bank criteria. Control participants are required to be in generally good health with no disorder that causes involuntary movements or gait disturbances.

The study protocol was reviewed and approved by the University of Rochester Research Subjects Review Boards (RSRB00001787); MIT Institutional Review Board (IRB) ceded to the Rochester IRB. The participants provided written informed consent to participate in this study.

#### MJFF dataset

The MJFF dataset is comprised of PD and normal participants who underwent an in-home observational study between 1 July 2018 and 1 January 2020. The inclusion criteria require participants to be able to speak and understand English, have capacity to provide informed consent, be ambulatory, have Wi-Fi in their residence, agree to allow for coded clinical research and for data to be shared with study collaborators, be willing and able to complete study activities, live with no more than one additional individual in the same residence and have no more than one ambulatory pet. The PD participants are required to have a diagnosis of PD according to the UK PD Brain Bank Criteria. Control participants are required to have no clinical evidence of PD, to not live with individuals with PD or other disorder that affects ambulatory status and to be age-matched to PD participants.

The study protocol was reviewed and approved by the University of Rochester Research Subjects Review Boards (RSRB00072169); MIT Institutional Review Board and Boston University Charles River Campus IRB ceded to the Rochester IRB. The participants provided written informed consent to participate in this study.

#### MIT dataset

The MIT control dataset is comprised of participants who underwent an in-home observational study between 1 June 2020 and 1 June 2021. The study investigates the use of wireless signals to monitor movements and vital signs. The inclusion criteria require participants to be above 18 years old, have home Wi-Fi, be able to give informed consent or have a legally authorized representative to provide consent, agree to confidential use and storage of all data and the use of all anonymized data for publication including scientific publication.

The study protocol was reviewed and approved by Massachusetts Institute of Technology Committee on the Use of Humans as Experimental Subjects (COUHES) (IRB no.: 1910000024). The participants provided written informed consent to participate in this study.

#### MGH dataset

The MGH control dataset is comprised of adult male and female subjects who have undergone in-lab PSG in the MGH for Sleep Medicine between 1 January 2019 and 1 July 2021. The MGH PD dataset is comprised of PD participants recruited from the Parkinson’s Disease and Movement Disorders Centers at Northwestern University and the Parkinson’s Disease and Movement Disorders Program at Rush University between 1 March 2007 and 31 October 2012. PD patients were enrolled in the study if they (1) had a diagnosis of idiopathic PD, as defined by the UK Parkinson’s Disease Society Brain Bank Criteria; (2) were classified as H&Y stages 2–4; (3) had EDS, as defined by an ESS score of 12 or greater; (4) had a stable PD medication regimen for at least 4 weeks before study screening; and (5) were willing and able to give written informed consent. Patients were excluded from participation if they (1) had atypical parkinsonian syndrome; (2) had substantial sleep-disordered breathing, defined as an apnea-hypopnea index of more than 15 events per hour of sleep on screening PSG; (3) had substantial periodic limb-movement disorder, defined as a periodic limb-movement arousal index of more than ten events per hour of sleep on screening PSG; (4) had REM sleep behavior disorder based on the presence of both clinical symptomatology and intermittent loss of REM atonia on screening PSG; (5) had cognitive impairment, as indicated by a Mini-Mental State Examination score less than 24; (6) had untreated hallucinations or psychosis; (7) used hypnosedative or stimulant drugs; (8) used antidepressants, unless the patient had been receiving a stable dose for at least 3 months; (9) had visual abnormalities that may interfere with light therapy (LT), such as significant cataracts, narrow-angle glaucoma or blindness; or (10) traveled across two or more time zones within 90 days before study screening.

The study protocols involving PD participants were reviewed and approved by the IRBs of Northwestern University, Rush University, and MGH. All study participants provided written informed consent. The protocol involving control participants and the sharing of deidentified data with MIT were reviewed by the Mass General Brigham IRB (IRB no. 2018P000337).

#### Mayo Clinic dataset

The Mayo Clinic PD dataset is comprised of adult subjects who underwent in-lab PSG between 1 January 2020 and 22 July 2021 and carried a diagnosis code for PD (ICD-10 CM G20 or ICD-9 CM 332.0) at the time of PSG. The control dataset consists of adult male and female subjects who have undergone in-lab PSG in the Mayo Clinic Center for Sleep Medicine between 1 January 2020 and 22 July 2021.

The use of the Mayo Clinic dataset and sharing of deidentified data with MIT was reviewed by the Mayo Clinic IRB, and the study was conducted in accordance with Institutional regulations and appropriate ethical oversight. Waiver of informed consent and waiver of HIPAA authorization were granted as the Mayo Clinic portion of the study involves only use of deidentified retrospective records and does not involve any direct contact with study participants.

### Data preprocessing

The datasets were divided into two groups. The first group comes from PSG sleep studies. Such studies use a breathing belt to record the subject’s breathing signals throughout the night. They also include EEG and sleep data. The PSG datasets are the SHHS^26^ (*n* = 2,630 nights from 2,630 subjects), MrOS^30^ (*n* = 3,883 nights from 2,875 subjects) and MGH (*n* = 223 nights from 155 subjects) sleep datasets. Further, an external PSG dataset from the Mayo Clinic (*n* = 1,920 nights from 1,920 subjects) was held back during the AI model development and serves as an independent test set. The second group of datasets collects nocturnal breathing in a contactless manner using a radio device developed by our team at MIT^27^. The data were collected by installing a low-power radio sensor in the subject’s bedroom, and analyzing the radio reflections from the environment to extract the subject’s breathing signal as described in our previous work^28,29^. This group includes the MJFF dataset (*n* = 526 nights from 15 subjects), the Udall dataset (*n* = 1,734 nights from 20 subjects) and the MIT dataset (*n* = 1,048 nights from 56 subjects). The wireless datasets have several nights per subject and information about PD severity such as MDS-UPDRS and/or H&Y stage^32^.

We processed the data to filter out nights shorter than 2 h. We also filter out nights where the breathing signal is distorted or nonexistent, which occurs when the person does not wear the breathing belt properly for breathing belt data, and when a source of interference (for example, fans or pets) exists near the subject for wireless data. We normalized the breathing signal from each night by clipping values larger than a particular range (we used [−6, +6]), subtracting the mean of the signal and dividing by the s.d. The resulting breathing signal is a one-dimensional (1D) time series $x \in R^{1 \times f_bT}$, with a sampling frequency ﻿$f_{\mathrm{b}}$ of 10 Hz, and a length of *T* s.

We use the following variables to determine whether a participant has PD: ‘Drugs used to treat Parkinson’s’ for SHHS and ‘Has a doctor or other healthcare provider ever told you that you had Parkinson’s disease?’ for MrOS. The other datasets explicitly report whether the person has PD and, for those who do have PD, they provided their MDS-UPDRS and H&Y stage.

In the experiments involving distinguishing PD from AD, we use the following variables to identify AD patients: ‘Acetylcholine Esterase Inhibitors For Alzheimer’s’ for SHHS, and ‘Has a doctor or other healthcare provider ever told you that you had dementia or Alzheimer’s disease?’ for MrOS.

Photos of the radio device and breathing belt are in Extended Data Fig. 1. Consent was obtained from all individuals whose images are shown in Extended Data Fig. 1 for publication of these images.

### Sensing breathing using radio signals

By capturing breathing signals using radio signals, our system can run in a completely contactless manner. We leveraged past work on extracting breathing signals from radio frequency (RF) signals that bounce off people’s bodies. The RF data were collected using a multi-antenna frequency-modulated continuous waves (FMCW) radio, used commonly in passive health monitoring^28,29^. The radio sweeps the frequencies from 5.4 GHz to 7.2 GHz, transmits at submilliwatt power in accordance with Federal Communications Commission regulations and captures reflections from the environment. The radio reflections are processed to infer the subject’s breathing signals. Past work shows that respiration signals extracted in this manner are highly accurate, even when several people sleep in the same bed^27,28,45^. In this paper, we extract the participant’s breathing signal from the RF signal using the method developed by Yue et al.^28^, which has been shown to work well even in the presence of bed partners, producing an average correlation 0.914 with a United States Food and Drug Administration-approved breathing belt on the person’s chest. We further confirmed the accuracy of the results in a diverse population by collecting wireless signals and breathing belt data from 326 subjects attending the MGH sleep lab, and running the above method to extract breathing signals from RF signals. The RF-based breathing signals have an average correlation of 0.91 with the signals from a breathing belt on the subject’s chest.

### AI-based model

**Extended Data Fig. 9:**
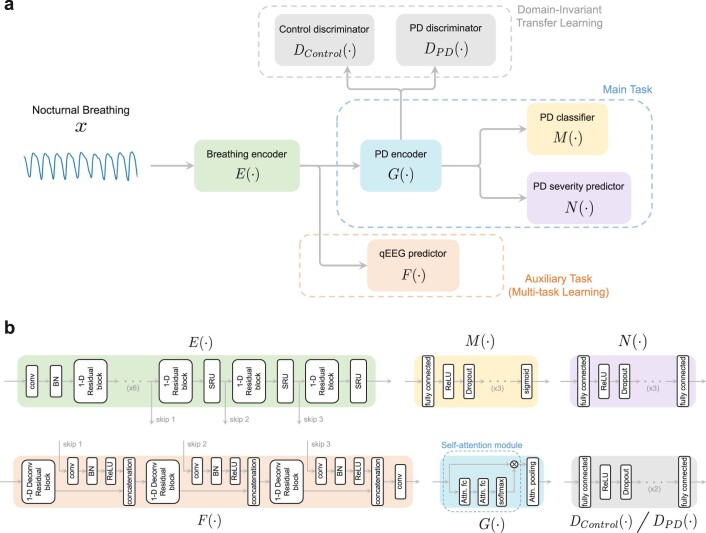
Neural network architecture of the AI-based model. **a**, The neural network takes as input a night of nocturnal breathing. The main task of PD prediction consists of a breathing encoder, a PD encoder, a PD classifier and a PD severity predictor. We also introduce an auxiliary task of predicting the subject’s qEEG during sleep. We include also two discriminators for domain-invariant transfer learning. **b**, The detailed architecture of each neural network module in the model.

We use a neural network to predict whether a subject has PD, and the severity of their PD in terms of the MDS-UPDRS. The neural network takes as input a night of nocturnal breathing. The neural network consists of a breathing encoder, a PD encoder, a PD classifier and a PD severity predictor (Extended Data Fig. 9).

Breathing encoder. We first used a breathing encoder to capture the temporal information in breathing signals. The encoder $E( \cdot )$ uses eight layers of 1D bottleneck residual blocks^33^, followed by three layers of simple recurrent units (SRU)^46^.

PD encoder. We then used a PD encoder to aggregate the temporal breathing features into a global feature representation. The PD encoder $G( \cdot )$ is a self-attention network^33^. It feeds the breathing features into two convolution layers with a stride of one followed by a normalization layer to generate the attention scores for each breathing feature. It then calculates the time average of the breathing features weighted by the corresponding attention scores as the global PD feature $G(E(x)) \in R^{d \times 1}$, where *d* is the fixed dimension of the global feature.

PD classifier: The PD classifier $M( \cdot )$ is composed of three fully connected layers and one sigmoid layer. The classifier outputs the PD diagnosis score *M*(*G*(*E*(*x*)), which is a number between zero and one. The person is considered to have PD if the score exceeds 0.5.

PD severity predictor. The PD severity predictor $N( \cdot )$ is composed of four fully connected layers. It outputs the PD severity estimation $N\left( {G(E(x))} \right.$, which is an estimate of the subject’s MDS-UPDRS score.

#### Multitask learning

To tackle the sparse supervision from PD labels (that is, only one label for around 10 h of nocturnal breathing signals), we introduce an auxiliary task of predicting a summary of the patient’s qEEG during sleep. The auxiliary task provides additional labels (from the qEEG signal) that help regularize the model during training. We chose qEEG prediction as our auxiliary task because EEG is related to both PD^37,38^ and breathing^47^. The datasets collected during sleep studies have EEG signals, making the labels accessible.

To generate the qEEG label, we first transform the ground-truth time series EEG signals into the frequency domain using the short-time Fourier transform and Welch’s periodogram method^48^. We extract the time series EEG signals from the C4-M1 channel, which is commonly used and available in sleep studies^26,30^. We then decompose the EEG spectrogram into the *Δ* (0.5–4 Hz), *θ* (4–8 Hz), *ɑ* (8–13 Hz) and *β* (13–30 Hz) bands^37–39^, and normalize the power to obtain the relative power in each band every second.

qEEG predictor. The qEEG predictor $F( \cdot )$, which takes as input the encoded breathing signals, and predicts the relative power in each EEG band at that time, consists of three layers of 1D deconvolution blocks, which upsample the extracted breathing features to the same time resolution as the qEEG signal, and two fully connected layers. Each 1D deconvolution block contains three deconvolution layers followed by batch normalization, rectified linear unit activation and a residual connection. We also used a skip connection by concatenating the output of SRU layers in the breathing encoder to the deconvolution layers in the qEEG predictor, which follows the UNet structure^33,49^. The predicted qEEG is $F(E(x))$.

#### Transfer learning

Our model leverages transfer learning to enable a unified model that works with both a breathing belt and a contactless radio sensor of breathing signals, and transfers the knowledge between different datasets.

Domain-invariant transfer learning. Note that our breathing signals are extracted from both breathing belts and wireless signals. There could exist a domain gap between these two data types, which makes jointly learning both of them less effective. To deal with this issue, we adversarially train the breathing encoder to ensure that the latent representation is domain invariant^50^. Specifically, we introduce a discriminator $D_{{\mathrm{PD}}}( \cdot )$ that differentiates features of breathing belt from features of wireless signals for PD patients. We then add an adversarial loss to the breathing encoder that makes the features indistinguishable by $D_{\mathrm{PD}}( \cdot )$. Similarly, we introduce a second discriminator $D_{\mathrm{Control}}( \cdot )$ with a corresponding adversarial loss for control subjects. We use two discriminators because the ratio of PD to control individuals is widely different between the wireless datasets and the breathing belt datasets (59% of the wireless data are from individuals with PD, whereas less than 2% of the breathing belt data comes from individuals with PD). If one uses a single discriminator, the discriminator may end up eliminating some features related to PD as it tries to eliminate the domain gap between the wireless dataset and the breathing-belt dataset.

Transductive consistency regularization. For PD severity prediction (that is, predicting the MDS-UPDRS), since we have several nights for each subject, the final PD severity prediction for each subject can further leverage the information that PD severity does not change over a short period (for example, 1 month). Therefore, the prediction for one subject across different nights should be consistent, that is, the PD severity prediction for different nights should be the same. To enforce this consistency, we add a consistency loss on the predictions of different nights (samples) for the same subject.

#### Distribution calibration

Since the percentage of individuals with PD is quite different between the wireless data and breathing belt data, we further calibrate the output probability of the PD classifier $M( \cdot )$ to ensure that all data types have the same threshold for PD diagnosis (that is, 0.5). Specifically, during training, we split training samples randomly into four subsets of equal size, and used three of them for training and the remaining one for calibration. We applied Platt Scaling^51^ to calibrate the predicted probability for PD diagnosis. After training a model using three subsets, we used the remaining calibration subset to learn two scalars $A,B \in R$ and calibrate the model output by $\hat y_c = \sigma (A\hat y + B)$, where $\hat y$ is the original model output, $\hat y_{\mathrm{c}}$ is the calibrated result and $\sigma ( \cdot )$ is a sigmoid function^52^. The cross-entropy loss between $\hat y_{\mathrm{c}}$ and *y* is minimized in the calibration subset. This process is repeated four times, with each subset used once for calibration, leading to four calibrated models. Our final model is the average ensemble of these models.

#### Training details

At each epoch, we randomly sampled a full-night nocturnal breathing signal as a mini-batch of the input. The total loss in general contains a weighted cross-entropy loss of PD classification, a weighted regression loss of MDS-UPDRS regression, an L2 loss of qEEG prediction, a discriminator loss of which domain the input comes from and a transductive consistency loss of minimizing the difference of the severity prediction across all nights from the same subject. For each specific input nocturnal breathing signal, total loss depends on the existing labels for that night. If one kind of label is not available, the corresponding loss term was excluded from the total loss. During training, the weights of the model were randomly initialized, and we used Adam optimizer^33^ with a learning rate of 1 × 10^−4^. The neural network model is trained on several NVIDIA TITAN Xp graphical processing units using the PyTorch deep learning library.

A detailed reporting of the AI model evaluation is provided in Supplementary Note 1.

### Details of the machine learning baselines used for comparison

We compared our model to the following machine learning baselines:

- We considered Support Vector Machine (SVM)^40^, which is used widely in the medical literature^53^. SVM can be used for both classification (that is, PD detection) and regression (that is, PD severity prediction) tasks. Since the input breathing signal is a time series, as common with SVM, we use principal component analysis to reduce the input dimension to 1,000.
- We also considered a basic neural network architecture that combines ResNet and LSTM. Such an architecture has been used in past work for learning from physiological signals^41^. The ResNet^33^ blocks use 1D convolution to encode the high-dimensional breathing into fixed-length feature vectors, which are then passed to LSTM modules^52^ for temporal understanding. The output of the network consists of two branches, one for PD detection and another for MDS-UPDRS prediction.

### Statistical analysis

#### PD diagnosis and PD severity prediction

Intraclass correlation coefficient (ICC) was used to assess test–retest reliability for both PD diagnosis and PD severity prediction. To evaluate PD severity prediction, we assessed the correlation between our model predictions (median value from all nights used) and clinical PD outcome measures (MDS-UPDRS total score) at the baseline visit using a Pearson correlation. We further compared the aggregated mean values among groups with different H&Y stages using the Kruskal–Wallis test (*α* = 0.05).

#### Risk assessments before clinical diagnosis

We assessed the capability of our AI-based system to identify high-risk individuals before actual diagnosis. For PD diagnosis, we compared the aggregated predictions between the prodromal group and the control group using the one-tailed Wilcoxon rank-sum test (*α* = 0.05). For PD severity prediction, we again used the one-tailed Wilcoxon rank-sum test (*α* = 0.05) to assess the PD severity prediction between the prodromal group and the control group.

#### Longitudinal disease progression analysis

We evaluated AI model predictions on disease severity across longitudinal data. To assess disease progression over 1 year, we aggregated the 1-year MDS-UPDRS change values over all patients, and used one-tailed one-sample Wilcoxon signed-rank test (*α* = 0.05) to assess the significance of 6-month and 12-month MDS-UPDRS change for both clinician assessment and our model prediction. For continuous severity prediction across 1 year, we further compared the aggregated model predictions with an interval length of 1 month using the Kruskal–Wallis test (*α* = 0.05).

#### qEEG and sleep statistics comparison between PD and control subjects

Finally, we assessed the distribution difference between control and PD subjects using an aggregate attention score associated with different EEG bands and sleep status. To do so, we used a one-tailed Wilcoxon rank-sum test (*α* = 0.05) for statistical analysis between the PD group and the control group.

All statistical analyses were performed with Python v.3.7 (Python Software Foundation) and R v.3.6 (R Foundation).

### Evaluation methods

To evaluate the performance of PD severity prediction, we use the Pearson correlation, which is calculated as:

$${\mathrm{Pearson}}\;{\mathrm{correlation}} = \frac{{\mathop {\sum }\nolimits_{{i} = 1}^N (u_{i} - \bar u)(v_{i} - \bar v)}}{{\sqrt {\mathop {\sum }\nolimits_{{i} = 1}^N (u_{i} - \bar u)^2} \sqrt {\mathop {\sum }\nolimits_{\mathrm{i} = 1}^N (v_{i} - \bar v)^2} }}$$

where *N* is the number of samples, *u*_*i*_ is the ground-truth MDS-UPDRS of *i*th sample, $\bar u$ is the average of all ground-truth MDS-UPDRS values, *v*_*i*_ is PD severity prediction of the *i*th sample and $\bar v$ is the average of all PD severity predictions.

To evaluate the performance of PD classification, we used sensitivity, specificity, ROC curves and AUC. Sensitivity and specificity were calculated as:

$$\mathrm{Sensitivity} = \frac{{\mathrm{TP}}}{{\mathrm{TP} + \mathrm{FN}}}$$

$$\mathrm{Specificity} = \frac{{\mathrm{TN}}}{{\mathrm{TN} + \mathrm{FP}}}$$

where TP is true positive, FN is false negative, TN is true negative and FP is false positive. When reporting the sensitivity and specificity, we used a classification threshold of 0.5 for both data from breathing belt and data from wireless signals. We followed standard procedures to calculate the 95% CI for sensitivity and specificity^54^.

We also evaluated the test–retest reliability. This is a common test for identifying the lower bound on the amount of data aggregation necessary to achieve a desirable statistical confidence in the repeatability of the result. The test–retest reliability was evaluated using the ICC^31^. To compute the ICC, we divided the longitudinal data into time windows. We use the month immediately after the baseline visit. Using more than a month of data is undesirable since a key requirement for test–retest reliability analysis is that, for each patient, the disease severity and symptoms have not changed during the period included in the analysis. We choose 1 month because this period is short enough to assume that the disease has not changed, and long enough to analyze various time windows for assessing reliability. From that period, we include all available nights. The ICC is computed as described by Guttman^31^.

### Reporting summary

Further information on research design is available in the Nature Research Reporting Summary linked to this article.

## Online content

Any methods, additional references, Nature Research reporting summaries, source data, extended data, supplementary information, acknowledgements, peer review information; details of author contributions and competing interests; and statements of data and code availability are available at 10.1038/s41591-022-01932-x.

### Supplementary information

Supplementary Information Supplementary Fig. 1 and Note 1.

Reporting Summary

## Figures and Tables

**Extended Data Table 1:**
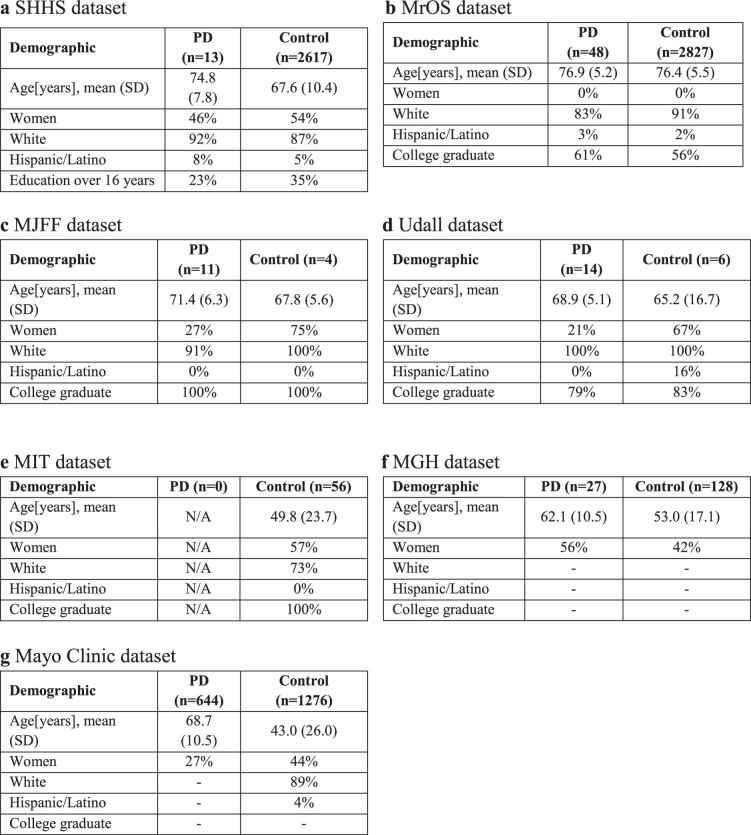
Demographic characteristics for each of the datasets used in this study

Hyphens indicate fields with unavailable data; N/A indicates fields for which the data are inapplicable.

## Data Availability

The SHHS and MrOS datasets are publicly available from the National Sleep Research Resource (SHHS: https://sleepdata.org/datasets/shhs; MrOS: https://sleepdata.org/datasets/mros). Restrictions apply to the availability of the in-house and external data (that is, Udall dataset, MJFF dataset, MIT dataset, MGH dataset and Mayo Clinic dataset), which were used with institutional permission through IRB approval, and are thus not publicly available. Please email all requests for academic use of raw and processed data to pd-breathing@mit.edu. Requests will be evaluated based on institutional and departmental policies to determine whether the data requested is subject to intellectual property or patient privacy obligations. Data can only be shared for noncommercial academic purposes and will require a formal data use agreement.

## Code availability

Code that supports the findings of this study will be available for noncommercial academic purposes and will require a formal code use agreement. Please contact pd-breathing@mit.edu for access.
