# Supplementary material for: Artificial intelligence-enabled detection and assessment of Parkinson’s disease using nocturnal breathing signals
Source: Nat Med. 2022 Aug 22;28(10):2207–15. doi: 10.1038/s41591-022-01932-x (PMC9556299; doi:10.1038/s41591-022-01932-x)
Supplement: Supplementary file 1 — Supplementary Fig. 1 and Note 1. [file 41591_2022_1932_MOESM1_ESM.pdf]

nature portfolio

https://doi.org/10.1038/s41591-022-01932-x

Supplementary information

Artificial intelligence-enabled detection and assessment of Parkinson's disease using nocturnal breathing signals

In the format provided by the authors and unedited

Supplementary Information

Supplementary Figure 1. Quantitative results on qEEG prediction from nocturnal breathing signals. We show the mean absolute error (MAE), and mean absolute percentage error (MAPE) metrics for predicting EEG across different frequency bands. MAE is defined as averaged absolute difference between the ground truth and predicted values over all samples. MAPE is defined as averaged absolute difference divided by ground truth over all samples. The results are computed for the PSG datasets which include EEG signals (n=6,660 nights from 5,652 subjects). The results show that the percentage errors in predicting qEEG varies between 10% to 30%, i.e., the accuracy varies between 90% and 70% depending on the qEEG band.

	Delta	Theta	Alpha	Beta	All
MAE	0.1211 ± 0.0316	0.0564 ± 0.0209	0.0552 ± 0.0190	0.0564 ± 0.0247	0.0723 ± 0.0172
MAPE (%)	10.485 ± 0.387	20.493 ± 2.885	29.429 ± 5.358	33.603 ± 6.071	21.222 ± 5.175

Supplementary Note 1. Checklist for supervised clinical ML study

Before paper submission			
**Study design (Part 1)**	**Completed: page number**		**Notes if not completed**
The clinical problem in which the model will be employed is clearly detailed in the paper.	☑	2, 3	
The research question is clearly stated.	☑	2, 3	
The characteristics of the cohorts (training and test sets) are detailed in the text.	☑	6, 7, 8, 24, 25, 26, 27, 28, 38	
The cohorts (training and test sets) are shown to be representative of real-world clinical settings.	☑	6, 7, 8, 24, 25, 26, 27, 28, 38	
The state-of-the-art solution used as a baseline for comparison has been identified and detailed.	☑	11, 12, 13, 14, 15, 16, 17	Note: There is no AI baseline that assesses PD from breathing. Thus for a baseline, we compare to the gold-standard in assessing PD, which is the MDS-UPDRS.
**Data and optimization (Parts 2, 3)**	**Completed: page number**		**Notes if not completed**
The origin of the data is described and the original format is detailed in the paper.	☑	24, 25, 26, 27, 28, 38	
Transformations of the data before it is applied to the proposed model are described.	☑	28, 29	
The independence between training and test sets has been proven in the paper.	☑	8, 9, 10, 11, 28, 34	
Details on the models that were evaluated and the code developed to select the best model are provided.	☑	34	
Is the input data type structured or unstructured?	☐ Structured ☑ Unstructured		
**Model performance (Part 4)**	**Completed: page number**		**Notes if not completed**
The primary metric selected to evaluate algorithm performance (eg: AUC, F-score, etc) including the justification for selection, has been clearly stated.	☑	36, 37	
The primary metric selected to evaluate the clinical utility of the model (eg PPV, NNT, etc) including the justification for selection, has been clearly stated.	☑	36, 37	

The performance comparison between baseline and proposed model is presented with the appropriate statistical significance.	☑	11, 12, 13, 14, 15, 16, 17, 35, 36	
**Model Examination (Parts 5)**	**Completed: page number**		**Notes if not completed**
Examination Technique 1[a]	☑	10, 13, 36, 37	
Examination Technique 2[a]	☑	18, 19, 20, 41	
A discussion of the relevance of the examination results with respect to model/algorithm performance is presented.	☑	8, 9, 10, 11, 12, 13, 14, 15, 16, 17	
A discussion of the feasibility and significance of model interpretability at the case level if examination methods are uninterpretable is presented.	☑	18, 19, 20, 41	
A discussion of the reliability and robustness of the model as the underlying data distribution shifts is included.	☑	9, 10, 11	
**Reproducibility (Part 6): choose appropriate tier of transparency**			**Notes**
Tier 1: complete sharing of the code		☑	Code that supports the findings of this study will be available for non-commercial academic purposes and will require a formal code use agreement. Please contact pd-breathing@mit.edu for access.
Tier 2: allow a third party to evaluate the code for accuracy/fairness; share the results of this evaluation		☐	
Tier 3: release of a virtual machine (binary) for running the code on new data without sharing its details		☐	
Tier 4: no sharing		☐	

PPV: Positive Predictive Value
NNT: Numbers Needed to Treat

[a] Common examination approaches based on study type: for studies involving exclusively structured data, coefficients and sensitivity analysis are often appropriate; for studies involving unstructured data in the domains of image analysis or natural language processing, saliency maps (or equivalents) and sensitivity analyses are often appropriate. Select 2 from this list or chose an appropriate technique, document each technique used on the appropriate line above.
