# Supplementary material for: Artificial intelligence-enabled detection and assessment of Parkinson’s disease using nocturnal breathing signals
Source: Nat Med. 2022 Aug 22;28(10):2207–15. doi: 10.1038/s41591-022-01932-x (PMC9556299; doi:10.1038/s41591-022-01932-x)
Supplement: Supplementary file 2 — Reporting Summary [file 41591_2022_1932_MOESM2_ESM.pdf]

nature portfolio

Corresponding author(s): Yuzhe Yang, Yuan Yuan

Last updated by author(s): Jul 1, 2022

Reporting Summary

Nature Portfolio wishes to improve the reproducibility of the work that we publish. This form provides structure for consistency and transparency in reporting. For further information on Nature Portfolio policies, see our Editorial Policies and the Editorial Policy Checklist.

Statistics

For all statistical analyses, confirm that the following items are present in the figure legend, table legend, main text, or Methods section.

n/a	Confirmed	
☐	☒	The exact sample size (*n*) for each experimental group/condition, given as a discrete number and unit of measurement
☐	☒	A statement on whether measurements were taken from distinct samples or whether the same sample was measured repeatedly
☐	☒	The statistical test(s) used AND whether they are one- or two-sided<br>*Only common tests should be described solely by name; describe more complex techniques in the Methods section.*
☐	☒	A description of all covariates tested
☐	☒	A description of any assumptions or corrections, such as tests of normality and adjustment for multiple comparisons
☐	☒	A full description of the statistical parameters including central tendency (e.g. means) or other basic estimates (e.g. regression coefficient) AND variation (e.g. standard deviation) or associated estimates of uncertainty (e.g. confidence intervals)
☐	☒	For null hypothesis testing, the test statistic (e.g. *F*, *t*, *r*) with confidence intervals, effect sizes, degrees of freedom and *P* value noted<br>*Give P values as exact values whenever suitable.*
☒	☐	For Bayesian analysis, information on the choice of priors and Markov chain Monte Carlo settings
☒	☐	For hierarchical and complex designs, identification of the appropriate level for tests and full reporting of outcomes
☐	☒	Estimates of effect sizes (e.g. Cohen's *d*, Pearson's *r*), indicating how they were calculated

Our web collection on statistics for biologists contains articles on many of the points above.

Software and code

Policy information about availability of computer code

Data collection	Code was written in Python version 3.7 (Python Software Foundation), PyTorch version 1.0.1, and R version 3.6 (R Foundation) and is available for non-commercial research purpose upon request from the authors.
Data analysis	Code was written using Python version 3.7 (Python Software Foundation), PyTorch version 1.0.1, and R version 3.6 (R Foundation). Code is available for non-commercial research purpose upon request from the authors.

For manuscripts utilizing custom algorithms or software that are central to the research but not yet described in published literature, software must be made available to editors and reviewers. We strongly encourage code deposition in a community repository (e.g. GitHub). See the Nature Portfolio guidelines for submitting code & software for further information.

Data

Policy information about availability of data

All manuscripts must include a data availability statement. This statement should provide the following information, where applicable:

- Accession codes, unique identifiers, or web links for publicly available datasets
- A description of any restrictions on data availability
- For clinical datasets or third party data, please ensure that the statement adheres to our policy

The SHHS and MrOS dataset are publicly available from the National Sleep Research Resource (SHHS: https://sleepdata.org/datasets/shhs; MrOS: https://sleepdata.org/datasets/mros). Restrictions apply to the availability of the in-house and external data (i.e., Udall dataset, MJFF dataset, MIT dataset, MGH dataset and Mayo Clinic dataset), which were used with institutional permission through IRB approval, and are thus not publicly available. Please email all requests for academic use of raw and processed data to pd-breathing@mit.edu. All requests will be evaluated based on institutional and departmental policies to determine

whether the data requested is subject to intellectual property or patient privacy obligations. Data can only be shared for non-commercial academic purposes and will require a formal data use agreement.

Field-specific reporting

Please select the one below that is the best fit for your research. If you are not sure, read the appropriate sections before making your selection.

☒ Life sciences ☐ Behavioural & social sciences ☐ Ecological, evolutionary & environmental sciences

For a reference copy of the document with all sections, see nature.com/documents/nr-reporting-summary-flat.pdf

Life sciences study design

All studies must disclose on these points even when the disclosure is negative.

Sample size

The dataset is created by pulling together multiple datasets from various medical centers including Mayo Clinic, MGH sleep lab, observational PD clinical trials such as the Michael J. Fox study, the Udall study, the MIT study, and public sleep datasets from the National Sleep Research Resource such as Sleep Heart Health Study (SHHS) and MrOS Sleep Study (MrOS). The combined dataset contains 11,964 nights with over 120,000 hours of nocturnal breathing signals from 757 PD subjects (mean (SD) age 69.1 (10.4), 27% women) and 6,914 control subjects (mean (SD) age 66.2 (18.3), 30% women). Data from Mayo Clinic (1,920) is held back during the AI model development, and serves as an independent test set. Further details and the demographic of the dataset are available in Methods and Extended Data Table 1.

Choice of Sample Size: For training and cross-validating the machine learning model, the choice of sample size was motivated by maximizing the size of the available dataset by pulling together multiple datasets as mentioned above. As is standard in the field, the data is divided into several subsets, where in each run, one subset is held-out for testing, and the rest are used for training so that the model is never trained and tested on the same dataset. For the other results in the paper, i.e., Figures 3, 4, and 5, the choice of sample size was to ensure statistical significance (i.e., $p < 0.05$).

Data exclusions

Data exclusion criteria includes:
1. Nights with breathing signal shorter than 2 hours.
2. Nights in which the breathing signal is non-existent or has significant white noise.
No other data were excluded.

Replication

Test retest reliability was used to ensure reliability and reproducibility. Please note that in the context of this paper, there is no notion of successful or unsuccessful replication. The difference between repetitions is not about whether they succeed or fail, it is about the variability in the output. Hence, the standard approach to characterize replication is by computing the test retest reliability and the associated confidence intervals, which were presented in Figures 2 and 3.

Randomization

The dataset was randomly split into training, validation, and test sets (60%/15%/25%), and this procedure was repeated 4 times to ensure all patients appeared in the test set once. The split was performed on a patient level, i.e., ensuring that no patient is both in testing and training in the same time.

Blinding

The test sets were held back during the AI system development, which only had access to the training and validation sets. Further, the full dataset from Mayo Clinic was held back during the model development, and served as an independent external test set. Investigators did not access test data until the model, the hyperparameters, and the thresholds were all finalized.

Reporting for specific materials, systems and methods

We require information from authors about some types of materials, experimental systems and methods used in many studies. Here, indicate whether each material, system or method listed is relevant to your study. If you are not sure if a list item applies to your research, read the appropriate section before selecting a response.

Materials & experimental systems

n/a	Involved in the study	
☒	☐	Antibodies
☒	☐	Eukaryotic cell lines
☒	☐	Palaeontology and archaeology
☒	☐	Animals and other organisms
☐	☒	Human research participants
☒	☐	Clinical data
☒	☐	Dual use research of concern

Methods

n/a	Involved in the study	
☒	☐	ChIP-seq
☒	☐	Flow cytometry
☒	☐	MRI-based neuroimaging

Human research participants

Policy information about studies involving human research participants

Population characteristics	The data contains 11,964 nights with over 120,000 hours of nocturnal breathing signals from 757 PD subjects (mean (SD) age 69.1 (10.4), 27% women) and 6,914 control subjects (mean (SD) age 66.2 (18.3), 30% women). The demographic information for each of the datasets are available in Extended Data Table 1.
Recruitment	Patients were not directly involved or recruited for the study. This study involved retrospective analysis of PD and non-PD breathing data collected during sleep studies which are either performed during standard clinical care, or are available publicly from the National Sleep Research Resource. All data was de-identified.
Ethics oversight	Study procedures were approved by the corresponding Institutional Review Boards (IRBs) at Mayo Clinic, MGH, MIT, and University of Rochester.<br><br>Udall dataset: The study protocol was reviewed and approved by the University of Rochester Research Subjects Review Boards (RSRB00001787); MIT institutional Review Board to the Rochester IRB. The participants provided their written informed consent to participate in this study.<br><br>Michael J. Fox (MJFF) dataset: The studies involving human participants were reviewed and approved by the University of Rochester Research Subjects Review Boards (RSRB00072169); MIT institutional Review Board and Boston University Charles River Campus Institutional Review Board ceded to the Rochester IRB. The participants provided their written informed consent to participate in this study.<br><br>MIT dataset: The studies involving human participants were reviewed and approved by Massachusetts Institute of Technology Committee on the Use of Humans as Experimental Subjects (COUHES) (IRB #: 1910000024). The participants provided their written informed consent to participate in this study.<br><br>MGH dataset. The study protocols involving PD participants were reviewed and approved by the institutional review boards of Northwestern University, Chicago, Illinois; Rush University, Chicago; and Massachusetts General Hospital, Boston, MA. All study participants provided written informed consent. The protocols involving control participants and the sharing of de-identified data with MIT was reviewed by the Mass General Brigham Institutional Review Board (IRB number 2018P000337).<br><br>Mayo Clinic dataset: The use of the Mayo Clinic dataset and sharing of de-identified data with MIT was reviewed by the Mayo Clinic Institutional Review Board and the study was conducted in accordance with Institutional regulations and appropriate ethical oversight. Waiver of informed consent and waiver of HIPAA authorization were granted as the Mayo Clinic portion of the study involves only use of de-identified retrospective records and does not involve any direct contact with study participants.

Note that full information on the approval of the study protocol must also be provided in the manuscript.
